# Dr. Charles A. Gorse

**Published:** 1912-11

**Authors:** 


Dr. Charles A. Gorse of Meadow Brook, Orange Co., was shot by his insane son, Oct. 10.

